# Smartphone-Based Ecological Momentary Assessment for the Measurement of the Performance Status and Health-Related Quality of Life in Cancer Patients Under Systemic Anticancer Therapies: Development and Acceptability of a Mobile App

**DOI:** 10.3389/fonc.2022.880430

**Published:** 2022-07-13

**Authors:** Vicente Escudero-Vilaplana, Lorena Romero-Medrano, Cristina Villanueva-Bueno, Marta Rodríguez de Diago, Alberto Yánez-Montesdeoca, Roberto Collado-Borrell, Juan José Campaña-Montes, Belén Marzal-Alfaro, José Luis Revuelta-Herrero, Antonio Calles, Mar Galera, Rosa Álvarez, Ana Herranz, María Sanjurjo, Antonio Artés-Rodríguez

**Affiliations:** ^1^ Pharmacy Department, Hospital General Universitario Gregorio Marañón, Madrid, Spain; ^2^ Instituto de Investigación Sanitaria Gregorio Marañón, Madrid, Spain; ^3^ Evidence-Based Behavior, Madrid, Spain; ^4^ Department of Signal Theory and Communications, Universidad Carlos III de Madrid, Madrid, Spain; ^5^ Medical Oncology Department, Hospital General Universitario Gregorio Marañón, Madrid, Spain; ^6^ Centro de Investigación Biomédica en Red de Salud Mental (CIBERSAM), Carlos III Institute of Health, Madrid, Spain

**Keywords:** app, patient-reported outcomes, quality of life, Eastern Cooperative Oncology Group (ECOG), machine learning, cancer, smartphone-based ecological momentary assessment

## Abstract

**Background:**

We have defined a project to develop a mobile app that continually records smartphone parameters which may help define the Eastern Cooperative Oncology Group performance status (ECOG-PS) and the health-related quality of life (HRQoL), without interaction with patients or professionals. This project is divided into 3 phases. Here we describe phase 1. The objective of this phase was to develop the app and assess its usability concerning patient characteristics, acceptability, and satisfaction.

**Methods:**

The app eB2-ECOG was developed and installed in the smartphone of cancer patients who will be followed for six months. Criteria inclusion were: age over 18-year-old; diagnosed with unresectable or metastatic lung cancer, gastrointestinal stromal tumor, sarcoma, or head and neck cancer; under systemic anticancer therapies; and possession of a Smartphone. The app will collect passive and active data from the patients while healthcare professionals will evaluate the ECOG-PS and HRQoL through conventional tools. Acceptability was assessed during the follow-up. Patients answered a satisfaction survey in the app between 3-6 months from their inclusion.

**Results:**

The app developed provides a system for continuously collecting, merging, and processing data related to patient’s health and physical activity. It provides a transparent capture service based on all the available data of a patient. Currently, 106 patients have been recruited. A total of 36 patients were excluded, most of them (21/36) due to technological reasons. We assessed 69 patients (53 lung cancer, 8 gastrointestinal stromal tumors, 5 sarcomas, and 3 head and neck cancer). Concerning app satisfaction, 70.4% (20/27) of patients found the app intuitive and easy to use, and 51.9% (17/27) of them said that the app helped them to improve and handle their problems better. Overall, 17 out of 27 patients [62.9%] were satisfied with the app, and 14 of them [51.8%] would recommend the app to other patients.

**Conclusions:**

We observed that the app’s acceptability and satisfaction were good, which is essential for the continuity of the project. In the subsequent phases, we will develop predictive models based on the collected information during this phase. We will validate the method and analyze the sensitivity of the automated results.

## 1 Introduction

There were an estimated 19.3 million new cases and 10 million cancer deaths worldwide in 2020 (1). Tumor characteristics as well as patient factors, especially performance status and quality of life, determine the choice of treatment. Despite significant advances in recent years in these therapies, thoracic and head and neck cancers are particularly associated with a higher burden of symptoms and impact on quality of life (2–4). Fatigue, dyspnea, coughing, loss of appetite, and pain have a significant effect on patients at psychological, emotional, and physical levels (5–8).

Patient-reported outcomes (PROs) are measurements of the patient’s direct perception of their health (9). The poor prognosis of some advanced cancer, the toxicity of the treatments, and the presence of comorbidities make PROs increasingly crucial in the decision-making process of this disease (10). The systematic and continuous evaluation of PROs has been associated with a higher quality of life and a higher survival rate in oncology patients (11, 12). However, collecting this type of results in these patients is limited to some research studies and is not frequent in clinical practice.

One of the main barriers in evaluating these PROs is the lack of standardized and validated tools for their follow-up. The International Consortium for Health Outcomes Measurements (ICHOM) has developed standard sets for several diseases, in which the main results that matter to patients and professionals are defined (13). In the case of the standard set for lung cancer, the measures to evaluate the PROs are the Eastern Cooperative Oncology Group performance status (ECOG-PS) and two questionnaires on health-related quality of life (HRQoL) (EORTC QLQ-LC30 and EORTC QLQ-LC13) (10). A Spanish adaptation of this standard set has proposed two other questionnaires (EQ-5D-5L –a generic questionnaire- and LCSS –a specific lung cancer questionnaire) as more efficient tools for assessing HRQoL in these patients, since the time required to complete them is much shorter (14). ICHOM is aware of the difficulty of implementing this methodology in clinical practice and its workload. Thus, to reach a more significant number of patients and reduce costs (10), ICHOM proposes to rely on information and communication technologies (ICT).

mHealth is revolutionizing the recording of health data since it allows collection at any location or time and the possibility of remote monitoring of patient activity (15). Smartphones are provided with different sensors like accelerometers, pedometers, gyroscopes, etc., becoming a rich data source to collect objective daily information from the user. The use of Wi-Fi and the advances in processing these data through accessible apps permit the collection of many variables derived from these base sensors in real-time: location, distance traveled, or the performance of physical activities (walking, running, sitting), among others, with application in several fields and, in particular, in health care (16, 17).

Within mHealth, we find Ecological Momentary Assessment (EMA). EMA is a collection of methods that allow us to collect data and capture information, using a repeated collection of experiences, cognitions, and behaviors as they happen (18). The collection of these data across multiple days and among various participants can provide health professionals with a profound insight into the studied daily life experience(s). To draw consistent conclusions from the data, it is necessary to collect these data passively (19). Additionally, these technologies, together with machine learning and data processing techniques, allow monitoring of patients without invading their privacy or affecting their daily lives (15, 20).

Our objective was the development of a mobile app that continuously and in an automated way evaluates and records the ECOG-PS and HRQoL of cancer patients. Additionally, we reported data on patients’ acceptability and satisfaction results.

## 2 Material and Methods

The research team is composed of oncologists belonging to a Thoracic Oncology, Head and Neck, and Sarcoma Unit; hospital pharmacists specialized in oncology; and engineers specialized in Signal Theory and Communications.

### 2.1 Study Design

The study project was divided into three phases. Here we describe the development of the system (phase 1).

**Figure 1:**
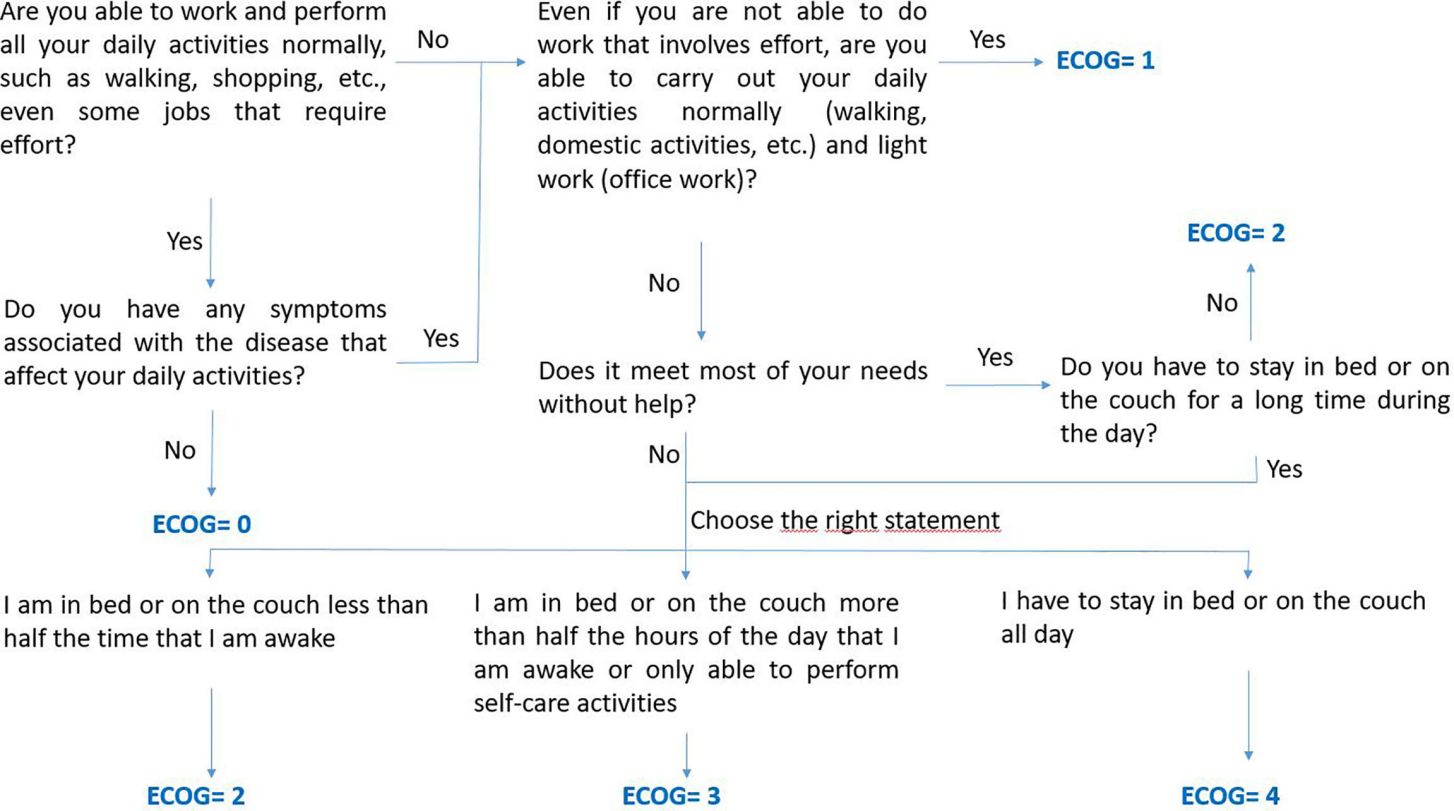
Algorithm defined by the study investigators to guide healthcare professionals on the questions to ask patient to calculate the ECOG-PS.

The research team, with prior experiences (21, 22), has developed a new app called eB2-ECOG for cancer patients. The app was developed in Spanish. Once developed and tested, the app has been installed in the patients’ smartphones included in this phase of the study. At the same time, healthcare professionals (pharmacists and oncologists) will evaluate the ECOG-PS and HRQoL through conventional tools. An algorithm was defined to calculate the ECOG-PS and reduce the variability among different professionals (**Figure 1**). Each patient underwent follow-up every 6 months or until death.

In phase 2, we will develop machine learning algorithms to predict the ECOG-PS and HRQoL in an automated way. In phase 3 we will validate these algorithms in clinical practice.

### 2.2 Study Population

#### 2.2.1 Eligibility Criteria

Inclusion criteria: Age over 18-year-old; diagnosed with unresectable or metastatic lung cancer, gastrointestinal stromal tumor, sarcoma, or head and neck cancer followed by the Oncology Department; treatment with systemic anticancer therapies; and had a smartphone with Android operating system (version 4.4 or higher) or iOS (version 10 or higher).

Exclusion criteria: Patients without access to a Wi-Fi network.

#### 2.2.2 Sample Size

Considering the large amount of data generated from continuous monitoring over 6 months (the number of daily data captured would be more than 1000), and based on a Markovian hypothesis, a sample size of 100 patients is enough to establish predictive values between the variables measured by the app and the ECOG-PS and HRQoL scores.

### 2.3 Study Variables

#### 2.3.1 Sociodemographic Variables

Date of birth, sex, level of education (no education or incomplete primary education, primary education, secondary education, and university education), and family support.

#### 2.3.2 Clinical Variables

- Tumor data: type of tumor, date of diagnosis, and clinical and pathological stage according to the American Joint Committee on Cancer staging system.
- Performance status (ECOG-PS) (23). An algorithm has been defined to guide healthcare professionals on the questions to ask patients to calculate the ECOG-PS (**Figure 1**).
- Health-related quality of life: generic questionnaire (European Quality of Life 5-dimensions questionnaire - EQ-5D-5L) (24) for all types of cancers and a specific questionnaire for lung cancer (Lung Cancer Symptom Scale - LCSS) (25). These questionnaires allow gathering information about the patient’s perspective on their physical and emotional functioning, fatigue, vitality, pain, cough, breathing difficulty, hemoptysis, and loss of appetite.
- Date of death, or last follow up determined.

#### 2.3.3 Pharmacotherapeutic Variables

Systemic anticancer therapy used: start and end dates, type of drug, dosage, and reasons for dose reductions or treatment suspensions.

#### 2.3.4 Variables Captured and Evaluated Directly by the App

- Physical activity: these data are recorded in static and movement and different positions (orthostatic, sedentary, and decubitus). It identifies if the patient is using the mobile phone, walking, running, or lying down.
- Steps: obtained from the phone pedometer.
- Nearby Wi-Fi and Bluetooth devices.
- GPS location: the location is recorded every 5 minutes. The data are stored as latitude and altitude offset from an unknown center and rotated on a random angle. Thus, the position obtained is relative and maintains the patient’s privacy.
- Google location: the available Google services are used to obtain information about the places near the patient’s position. This information is saved as the types of nearby locations and the probability that the subject is in each of them.
- Telephone activity: the call traffic made by the patient, the type of call (incoming/outgoing/missing), the duration of the calls, and the time to answer an incoming call will be stored.
- Voice analysis: access to the device’s microphone and the speaker is intended to detect silences, dictations, durations of both speech pitch, and interruptions during the conversation. There is no provision for storing the audio recording.
- Type of app used (games, news, social networks): data are collected over periods and the percentage of time spent on each app.
- Actigraphy: level of amount of movement in a 10-second interval.

#### 2.3.5 Behavioral Indicators

Two daily behavioral indicators are calculated using the steps, GPS location, and telephone usage.

- Behavior indicator: This variable is 1 if a new behavior has started this day and 0 otherwise.
- Stability indicator: This variable is 1 if the user is stable this day and 0 otherwise (26).

This computation is based on the pre-processing of these data and the usage of proprietary algorithms already integrated with the eB2 platform.

#### 2.3.6 Satisfaction

**Figure 2:**
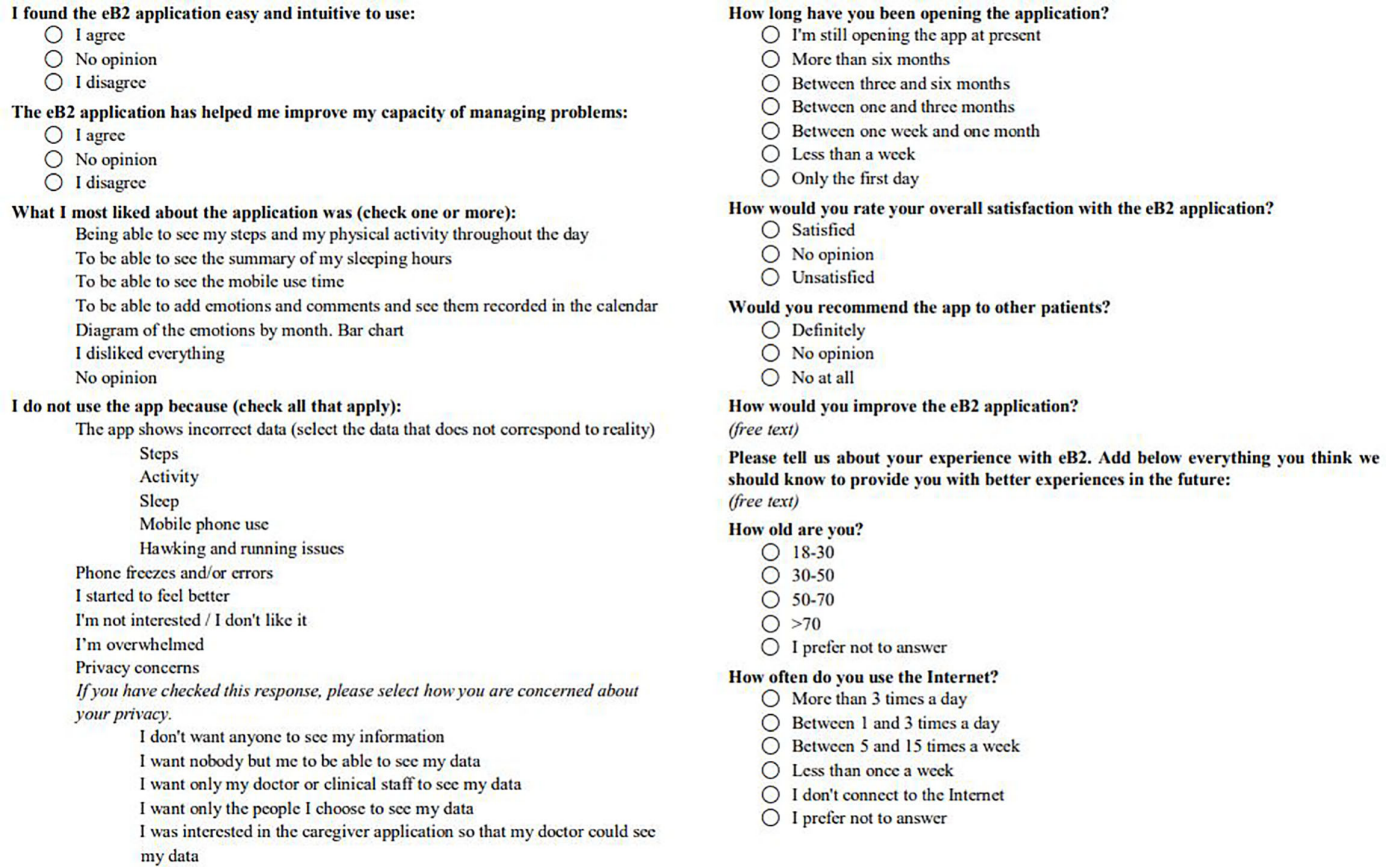
Patient app use and satisfaction questionnaire.

A survey for patients was included in the study (**Figure 2**). The satisfaction survey was applied *via* the app between 3-6 months from their inclusion.

### 2.4 Management, Data Privacy, and Ethics

#### Management

Patients were enrolled in the trial by oncologists or hospital pharmacists who also set up the app on the patient’s mobile phone during the medical or pharmaceutical consultation (27). The tool aims to provide a system for collecting, merging, and processing data related to the patient’s health and physical activity. It will provide a transparent capture service based on all the available data of a patient that will be merged to be treated by specific machine learning algorithms.

We have developed a web portal where oncologists and pharmacists can register patients and consult their status within the system. Information such as an anonymous patient ID, registration date, phone model, and operating system version is displayed. Besides, a color code reflects the patient status: green for correct uploaded data, yellow for pending data upload, and red for uninstalled app. If a total lack of activity is detected for more than 3 days, the health professional is altered.

#### Data privacy

The data evaluated by the health professionals will be registered in a REDCap (Research Electronic Data Capture) database. No personal data of patients will be registered on this platform. Patients will be identified with correlative numbers according to their inclusion. The collection, processing, and analysis of data will be carried out according to Regulation (EU) 2016/679 of the European Parliament and of the Council of 27 April 2016 on the protection of natural persons concerning the processing of personal data and the free movement of such data and repealing Directive 95/46/EC (General Data Protection Regulation).

The data recorded by the app is sent to a server that stores the information in a non-relational database. This server has secure communications protocols and bases its access policy on the OAuth2 scheme. The data are encrypted with a HASH SHA-1 function to preserve the anonymity of patients and their environment. The GPS location data will be anonymized from the app using a unique mobile device identifier (IMEI) and unknown to our system. Thus, it will not be possible to relate the location data to physical locations.

#### Ethics

The study was approved by the local Ethics Committee (Protocol code: PI17/02179) and was conducted under the ethical principles of the Declaration of Helsinki.

## 3 Results

### 3.1 App Functionalities

The app eB2-ECOG functionalities are mainly presented to the patient through five different screens: the home screen, activity screen, sleep screen, calendar screen, and emotion screen.

1. Home screen: On this first screen the users can see a general summary of their data. In the upper part of the screen, the total number of steps in the current day is shown jointly with the time performing different types of activity (walking, cycling, and vehicle). In the middle, sleep duration and sleep quality can be seen. In the bottom part, a summary of the emotions introduced along the day and the time using the phone are presented.
2. Activity screen: The users can check the time spent performing different activities and a personalized metric for the general level of activity of the day, obtained by comparing with the average level of activity of the users.
3. Sleep screen: A graphical representation of the sleep duration and quality over the week can be seen on this screen. Here, the duration and quality of sleep for the current day can be both validated or modified by the users.
4. Emotions screen: The users can actively record how they feel throughout the day by selecting a set of possible emotions.
5. Calendar screen: In the calendar, the users have an overview of the emotions recorded during the month and the particular history of a day.

**Figure 3:**
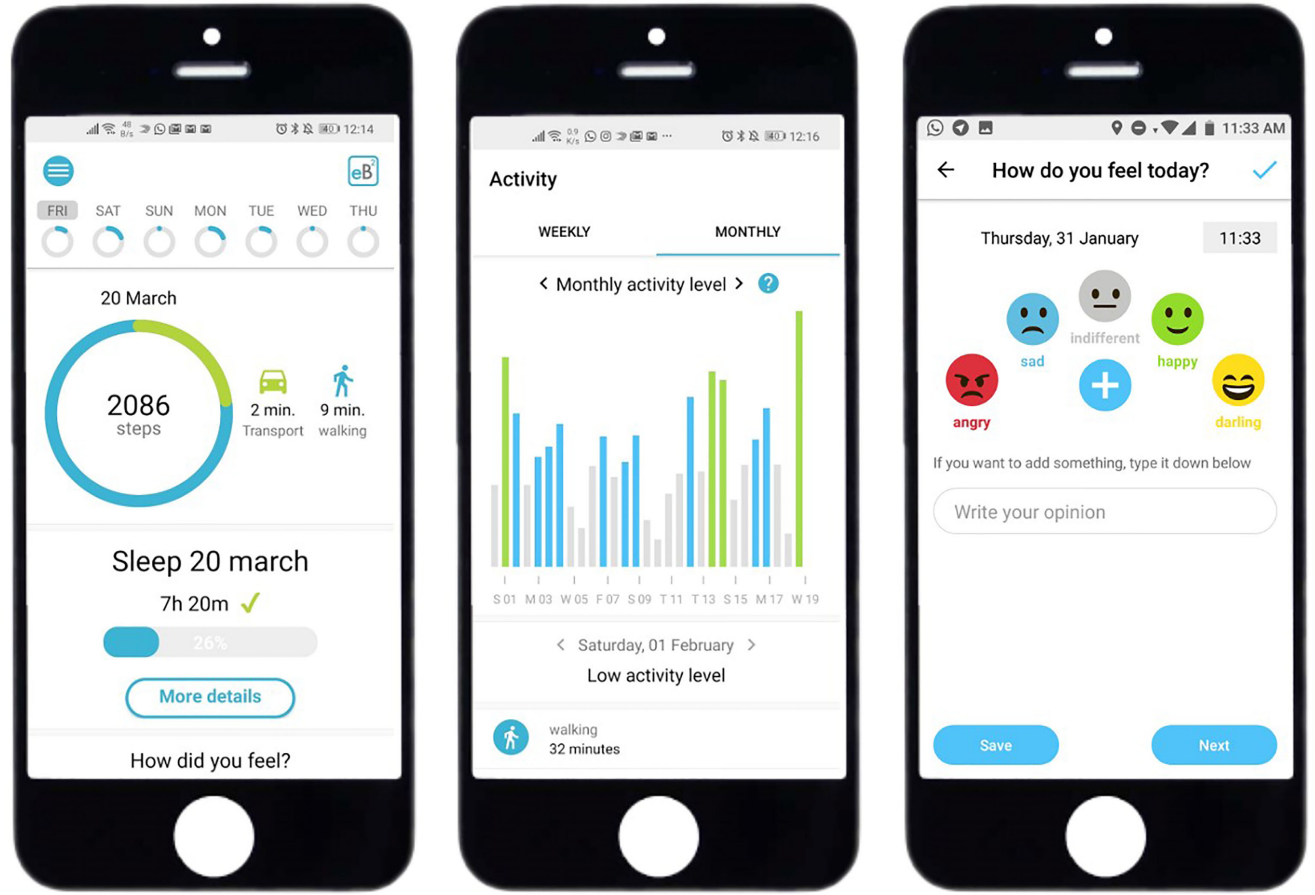
Diagram of the main mobile app screens.

In **Figure 3**, we show different the main important screens.

### 3.2 App Data Collection

#### User Registration and Activation

User registration is performed from the web portal by an authorized health professional. A non-related ID is created to identify each user. A one-time random 10-digit activation code is provided. The code is introduced in the mobile app to link the generated ID with the app user.

#### Permissions and Data Collection

After successful activation and registration, the user is introduced to the different features that the app provides. Permissions for data collection are asked for each of the collected variables previously mentioned. The rate of capturing passive data is every five minutes, and the uploading is performed every 2 hours if Wi-Fi is available. Otherwise, a data plan connection is used, but only with user permission. At the time of installation, the investigator ensured all relevant smartphone permissions were activated. In the case of active data as the case of emotions, the introduction is manual so the capture depends on the user. Once the app started, it runs in the background and can be easily accessed.

These passive data are collected from the phone sensor and optionally from wearable devices like activity trackers. The activity trackers that can be connected to the app are Fitbit and Garmin as specific wearables and Google Fit and Apple HealthKit as smartphone specific trackers. Some studies describe clinical validation of these wearables (28–30).

The collected data include sleep hours, movement collected with the accelerometer of the phone (time spent walking or moving by transport), visited places, use of other mobile applications, number of received and outgoing calls, and information regarding the voice tone (the conversations are not recorded, only the voice parameters are analyzed). To find useful clinical information all the collected data will be evaluated with machine learning techniques.

The app also allows the inserting of some active data (emotions). However, these data are not processed; it is only saved to help the patient carry a self-register if wanted.

### 3.3 Recruitment and Acceptability

**Figure 4:**
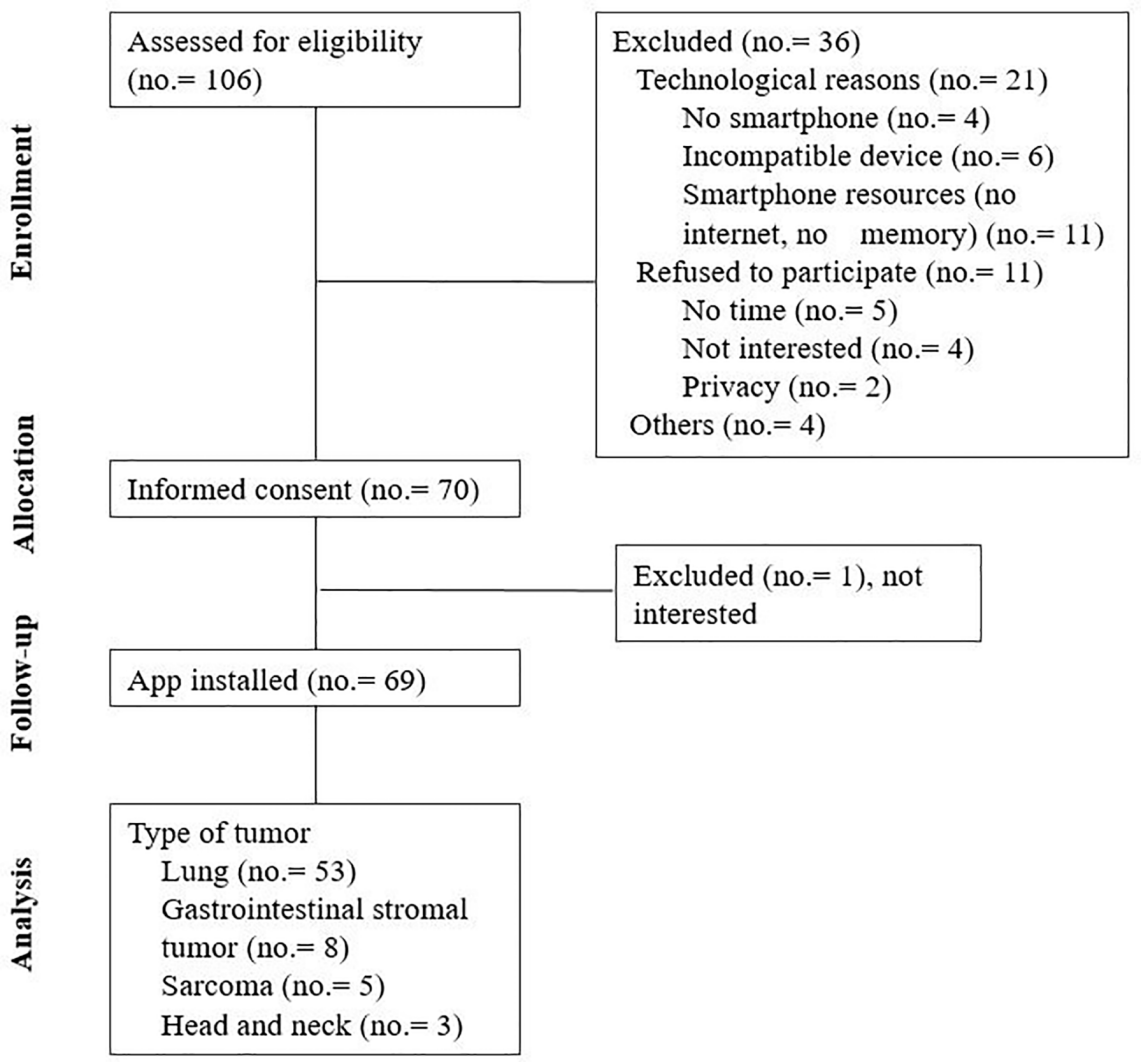
Acceptability: Phase 1 observational study diagram.

Currently, 106 patients have been recruited in this phase of the study (**Figure 4**).

The mean age of the population assessed for eligibility was 64.7 (range 27.3-89.9) years old, and 63.8% were male. The median age of the patients finally included was 61.4 (range 25.5-78.6) years, and the median age of the patient excluded was 72.4 (range 54.2-89.9) years. The difference in the median age among these two groups was 11.0 years (CI95% 8.3-18.0, p<0.001). No correlation was found between sex and acceptance to participate in the study (p=0.077). Among the patients excluded, the technological reasons for exclusion were also associated with older patients (p<0.001). The rest of the basal characteristics of the included patients are described in **Table 1**.

**Table 1:** Basal characteristics of the included patients.

Variables	Included patients, n = 69 (%)
Age (years)	61.4 (range 25.5-78.6)
Level of education	
No education or incomplete primary education Primary education Secondary education University education	0 (0%) 6 (8.7%) 26 (37.7%) 37 (53.6%)
Family support	69 (100%)
Type of tumor	
Lung Gastrointestinal stromal tumor Sarcoma Head and neck	53 (76.8%) 8 (11.6%) 5 (7.3%) 3 (4.3%)
ECOG-PS	
0 1 2	33 (47.8%) 34 (49.3%) 2 (2.9%)
Time since diagnosis (months)	15.8 (range 2.5-114.7)
Systemic anticancer therapy	
Chemotherapy Immunotherapy Chemoimmunotherapy Targeted therapy	33 (47.8%) 9 (13.0%) 5 (7.3%) 22 (31.9%)
No. of treatment line	
1 2 3 >3	36 (52.2%) 19 (27.5%) 7 (10.1%) 7 (10.1%)

**Figure 5:**
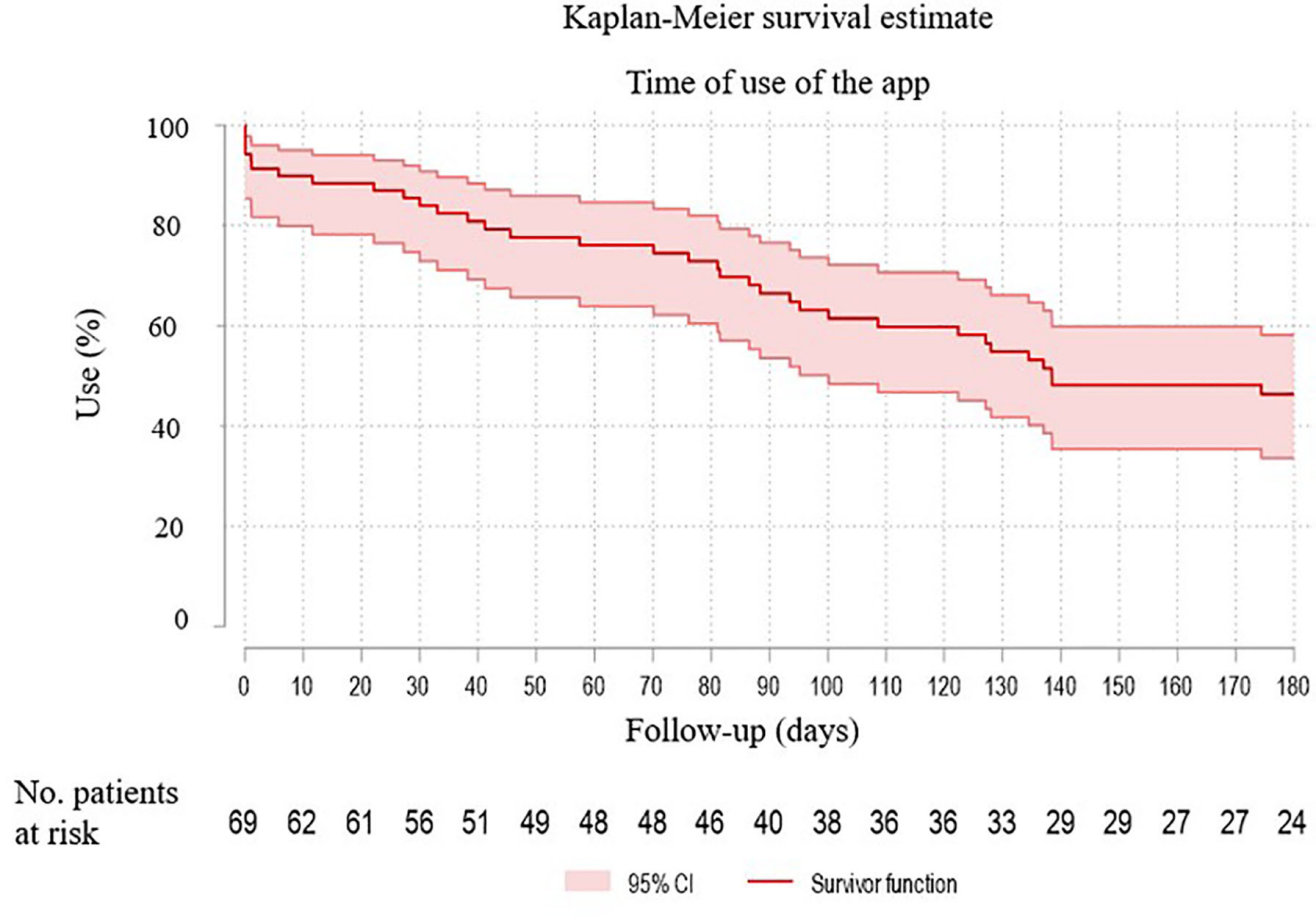
Kaplan-Meier survival estimates the use of the app during the follow-up.

The median follow-up time of patients using the app was 127 days (range 26-180) and, in the fourth month, 60% of users were still continuing. **Figure 5** estimates the use of the app during the follow-up.

### 3.4 Patient Satisfaction

Twenty-seven patients completed all the survey questions. The age ranges were as follows: 18-30 years old, 7.4% of patients; 30-50 years old, 11.1% of patients; 50-70 years old, 55.6% of patients; >70 years old, 11.1% of patients; and 14.8% of patients preferred not to answer. Concerning internet use, 40.7% of patients connected to the internet more than 3 times a day, 14.8% between 1 and 3 times a day, 14.8% between 5 and 15 times a week, 11.1% less than once a week, 11.1% did not connect to the intern frequently, and 7.4% preferred not to answer.

In total, 70.4% of patients found the app intuitive and easy to use and 51.9% said that the app helped them to improve and handle their problems better. 29.6% of patients used the app daily, 22.2% used the app last time less than a week ago, 7.4% between 1 week and 1 month, 7.4% between one and 3 months ago, 22.2% (6/27) between 3 and 6 months, 3.7% (1/27) more than 6 months ago, and 7.4% only used it on the first day.

The app’s features that patients liked the most were as follows: the step count and physical activity performed throughout the day (66.7% of patients); the summary of the sleeping hours (25.9%); and entry of emotions and comments and their records on the calendar (11.1%). 25.9% of patients did not usually use the app. The reasons why patients did not use it were as follows: the app misreported sleep data (28.6% of patients); the app was blocked or exited (28.6%); they felt overwhelmed (28.6%); and the app misreported mobile phone use data (14.3%). Overall, 62.9% of patients were satisfied with the app, and 51.8% would definitely recommend the app to other patients.

One-third of the patients (37.0%) gave their feedback about the app through the free-text area in the survey. Fifteen comments were collected, mainly focusing on their satisfaction with the app and areas for improvement. In general, the patient satisfaction was good (“the app is easy and intuitive to use”, “comprehensive app”, “very useful to know your daily activity”, “happy that my follow-up is improving”, etc.). Some comments on possible app improvements were as follows: “sometimes the sleep recording system is not accurate”, “it would be nice if it could be integrated with other similar apps so as not to have so many”, “it should be possible to include emotions retrospectively”, etc.

## 4 Discussion

### 4.1 Principal Findings and Comparison With Previous Studies

The systematic measurement of PROs in cancer patients (ECOG-PS and HRQoL) is a fundamental element in the management of these patients, since their degree of autonomy or dependence may determine the planning of treatment and care. We believe that the PROs and smartphone data that define the patient’s “digital phenotype”, captured passively (without the intervention of the patient or the professional) and systematically by our app, will be an adequate source of information to drive improved care for cancer patients.

The World Health Organization, regulatory agencies, and scientific societies recommend the evaluation and registration of the ECOG-PS of all cancer patients. Moreover, most clinical trials define the ECOG-PS as an inclusion/exclusion criterion (10, 31–34). ECOG-PS is considered a reliable predictor of survival and toxicity in patients with advanced cancer (35). Patients with poorer ECOG-PS usually need more palliative management, based mainly on support measures or less aggressive treatments, to avoid worsening quality of life. The use of systemic anticancer therapies at the end of life in patients with a medium-high ECOG-PS value does not improve quality of life (36). An experience at Cedars-Sinai Hospital in Los Angeles, which consisted of registering ECOG-PS on a mandatory basis in the prescription of systemic anticancer therapies, managed to reduce the use of these drugs in the last 14 days of life from 23% to 17% of patients (37). However, systematic evaluation and registration of ECOG-PS is not a standardized procedure in most centers. In a multicenter study involving 552 patients, 20% had no ECOG-PS values recorded in their clinical records (38). In other cases, the ECOG-PS was only recorded when starting a new systemic anticancer therapy; nevertheless, ECOG-PS is a parameter that evolves throughout the disease. Another limitation is the existence of biases due to the subjectivity of its measurement. The ECOG-PS in symptomatic patients or those with a poor general condition is something difficult to calculate, especially to the use of multiple parameters in the groupings. It has been observed that there is inter-observer variability in the assessment of performance status, with reduced sensitivity, especially in patients with poorer ECOG-PS (39, 40). To reduce this variability, we have defined an algorithm (**Figure 1**) to guide healthcare professionals on the questions to ask patients to calculate the ECOG-PS.

In addition to performance status, the overall assessment of the patient is completed with HRQoL scales. However, its systematic evaluation is not frequent in clinical practice (41), especially due to the time required to complete the questionnaires.

ICT can help overcome these limitations, as Memorial Sloan Kettering Cancer Center researchers recognize when they advocate for the evaluation of the predictive and prognostic capabilities of treatment outcomes through “electronic activity monitoring” (42). The aim is to make use of data on patients’ activity that are digitally available (“digital phenotype”) (20) to improve their diagnosis, follow-up, and treatment. The most efficient tool for these purposes is the use of mHealth (43). Integrating ICT into PROs evaluation is necessary to make their use a reality in daily practice. Otherwise, it will not go beyond the scope of research and clinical trials. These technologies must be developed to ensure accurate assessment of patient’s performance status and quality of life, which will help oncologists select the most appropriate therapy to reduce toxicity and improve outcomes (42). However, assessing patient satisfaction with these tools is essential for the success of methods to record PROs.

### 4.2 Comparison With Prior Work

There are currently more than 365,000 health apps (15), 40% of which are dedicated to patient care and monitoring. However, to the best of our knowledge, there is no app or tool available on the market that allows the evaluation of PROs in cancer patients in an automated way. Available apps to evaluate the quality of life in oncology patients are based merely on the integration of questionnaires into the app, that requires patient answering, with similar biases and problems to paper questionnaires (44). However, there are studies in psychiatry where the use of apps with technology similar to ours has contributed to better patient follow-up (19, 24, 45). In psychiatric patients, two leading indicators were found useful for health professionals: the change in behavior and the patient’s stability. The behaviors were defined by analyzing all the previously collected data in mobility, physical activity, and social activity. A behavior change was described as an abrupt and exaggerated change in usual activities and actions that a person has (46). This change can be related to a relapse of the illness and it can predict a worsening in the patient before the critical peak or even predict suicide attempts. This early detection allows health professionals to take actions more effectively and can lead to significant improvements in patients’ quality of life. The stability was related to the degree of change in the behavior and is a more visual way to see how critical is that change. If the difference is very abrupt and worrying, the patient can be considered “unstable”, and actions must be taken more urgently.

Continuous monitoring has aimed to identify behavioral changes in psychiatric patients and, thus, to anticipate new changes in their health status. In the field of oncology, studies have been conducted on tools that allow specific symptoms to be notified by patients in real-time, with an impact on the quality of life improvement and survival (11, 47). ASCO experts agree that it is impressive that something so simple not only improves the quality of life but helps patients live longer (42).

Many studies about mHealth services and methodological approaches show their promising use for several areas of health care, sometimes with advantages compared to the current standard approaches that might be either supplemented or replaced (16, 17). In the case of EMA, its use is frequently limited to mental health but its potential has started recently to be recognized also in cancer research (48). Kampshoff et al. presented a review of studies that apply active EMA techniques to address disease and drug-related problems among cancer patients. They found that EMA facilitated the assessment of real-time and real-scenario experiences and behavior (49). In our approach, we developed an automated app to continuously obtain the sensor data and PROs through personal smartphone monitoring and passive data collection from movement, physical, and social areas.

### 4.3 Limitations

Variability in the use of mobile phones among patients could alter some records, although due to previous initiatives developed this is not a significant problem (23, 24). The patient will be instructed orally and in writing on how to use the smartphone and the app. The requirement for the patient alone to install and use the app, and carry the smartphone makes the involvement of relatives and caregivers problematic as any involvement will lead to unrepresentative data.

If the app is not used, the automated activation of the battery manager could be another limitation in some smartphones. This activation may stop or slow down the data collection. However, these patients will be identified at the web portal as we described in the *Management, Data privacy, and Ethics* section.

In the case of the variables evaluated directly by the app, we mention a limitation regarding the physical activity records. This limitation is the true state of the patient in regards to being at rest or simply leaving the phone in a location. However, this is accounted for in the pre-processing. We check other variables when the physical activity variable indicates that the user has been lying down for a long period to know whether he/she has been using the phone for another purpose simultaneously, or check whether it is a usual sleeping time. If this is the case, we assume that the user was holding the smartphone without moving so, really lying down. If there is any doubt, the value is considered and treated as missing data for that period.

Concerning clinical limitations, we focused on thoracic, and head & neck cancer patients since they are tumors associated with a higher burden of symptoms and impact on quality of life. For tumors that produce many symptoms requiring frequent clinician involvement, or tumors which have very short survivals, the measurement of ECOG-PS and HRQoL will have much less utility.

### 4.4 Conclusions

We have developed an app whose final objective will be to continuously monitor in a non-intrusive way the ECOG-PS and HRQoL more efficiently for implementation in daily practice in cancer patients. In this first phase, we have observed that the app’s acceptability and satisfaction were good for our patients, which is essential for the continuation of the project. In the subsequent phases, we will validate the predictive value and analyze the sensitivity of the automated results obtained by the app.

## Data Availability Statement

The raw data supporting the conclusions of this article will be made available by the authors, without undue reservation.

## Ethics Statement

The studies involving human participants were reviewed and approved by Hospital General Universitario Gregorio Marañón. The patients/participants provided their written informed consent to participate in this study.

## Author Contributions

VE-V and LR-M had an equal contribution to the study and both wrote the first draft. VE-V, RC-B, AC, and AA-R defined the research question and objectives. LR-M, MD, AY-M, JC-M, and AA-R carried out the technical development of the app. VE-V, CV-B, RC-B, BM-A, JR-H, AC, MG, and RÁ included the patients and followed them up. AH, MS, and AA-R were responsible for the research activity plan and its execution. All authors contributed to the article and approved the submitted version.

## Funding

This work has been partly supported by the Spanish Ministerio de Ciencia, Innovación y Universidades (TEC2017-92552-EXP, RTI2018-099655-B-I00), the Comunidad de Madrid (Y2018/TCS-4705 PRACTICO-CM, IND2018/TIC-9649, IND2017/TIC-7618), and the BBVA Foundation (Deep-DARWiN grant).

## Conflict of Interest

The authors declare that the research was conducted in the absence of any commercial or financial relationships that could be construed as a potential conflict of interest.

## Publisher’s Note

All claims expressed in this article are solely those of the authors and do not necessarily represent those of their affiliated organizations, or those of the publisher, the editors and the reviewers. Any product that may be evaluated in this article, or claim that may be made by its manufacturer, is not guaranteed or endorsed by the publisher.
